# Associations of Prenatal Social Support in Adolescent Mothers With Measures of Social‐Emotional Development in Their Young Children

**DOI:** 10.1002/brb3.71059

**Published:** 2025-11-17

**Authors:** Cristin M. Holland, Huili Sun, Raimundo X. Rodriguez, Margaret Bennett, Bin Cheng, Bradley S. Peterson, Dustin Scheinost, Marisa N. Spann

**Affiliations:** ^1^ Vagelos College of Physicians and Surgeons Columbia University New York New York USA; ^2^ Yale University New Haven Connecticut USA; ^3^ Mailman School of Public Health Columbia University New York New York USA; ^4^ Keck School of Medicine University of Southern California Los Angeles California USA; ^5^ Children's Hospital Los Angeles Los Angeles California USA; ^6^ Yale School of Medicine New Haven Connecticut USA; ^7^ New York State Psychiatric Institute New York New York USA

**Keywords:** attachment, infant, language, maternal sensitivity, prenatal, social support

## Abstract

**Objective:**

Social environments are essential for supporting social‐emotional development in children. However, little consideration has been given to social environments children may be exposed to prior to birth and how this may affect social behavior for both generations postnatally. As such, this study considered the impact of maternal social support during pregnancy on infant and maternal social behaviors in very early childhood.

**Methods:**

Pregnant adolescents from 2009–2012 evaluated their social support and participated in maternal‐infant interactions at 4 and 14 months of age to assess maternal sensitivity and child attachment. Social skills and language development of infants were evaluated at 4 months of age. A subset of infants underwent MRI to assess functional connectivity of a social brain network. Path analyses were conducted to determine associations among prenatal maternal social support, 4‐month maternal sensitivity, 4‐month infant language and social development, and 14‐month child attachment.

**Results:**

We detected significant associations of prenatal social support satisfaction with 4‐month infant social development, and with maternal sensitivity and infant language development at 4 months. No maternal or infant factors were associated with attachment at 14 months. Exploratory path analysis included neonate social brain network connectivity. No significant associations were demonstrated between maternal or infant factors and neonate brain connectivity.

**Conclusion:**

This study underscores the importance of maternal perceived satisfaction with social support during pregnancy and sensitivity in infancy for child social and language development.

## Graphical abstract

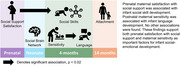

## 1 Introduction

Secure attachment in early years promotes self‐reliance, emotion regulation, and social competence in childhood and through adulthood (Cooke et al. 2019; Sroufe 2005; Sroufe et al. 2005). Foundations for social and emotional developmental trajectories leading to secure attachment are laid through early experiences that shape development in combination with genetics (Boyce et al. 2021). These experiences, including social and caregiving environments, can begin in utero. Recently, higher levels of social support were found to be a protective factor for pregnant women who reported depression and anxiety (Filippetti et al. 2022; Modde Epstein et al. 2024) and can ensure greater attachment to their fetus (Ponti et al. 2021). Social support may be an important factor in how bonding and interactive behavior with offspring may develop. A few studies identified that prenatal social support can buffer the effect of negative maternal mental health, including anxiety, depression, and stress on offspring's predictable social engagement during infancy (Takács et al. 2021) and attachment during toddler age (Jacobson and Frye 1991). However, research on social support during pregnancy and its role in the social‐emotional development of the child is still limited.

A more extensive literature exists demonstrating that, postnatally, infant social‐emotional development is influenced by caregiving behavior. Specifically, maternal sensitivity, the ability to detect and respond to infant cues, has been connected to social development and child attachment style. Caregiving behaviors that were more sensitive to the child's interest and did not control or restrict the child, predicted greater social development (Landry et al. 1997; Ziv et al. 2018). Higher sensitivity from mothers during infancy was positively associated with attachment at toddler and preschool ages (Bigelow et al. 2010; Braungart‐Rieker et al. 2014; Ziv et al. 2000), and may be a mechanism through which infant attachment security is formed when considering maternal embodied mentalizing, a parent's understanding of the thoughts, feelings, and attitudes of a child (Gagné et al. 2021). Additionally, higher and positive maternal responsiveness, which is also used to describe how mothers react to their children during dyadic interactions, was associated with greater social, emotional, and communication skills, as well as overall growth of social‐emotional competence (Bornstein and Manian 2013; Landry et al. 2006; Merz et al. 2015; Pearson et al. 2011).

Similar to social support, maternal sensitivity and responsiveness have been shown to buffer adverse experiences on offspring social‐emotional development (Wurster et al. 2020). For example, maternal sensitivity has been shown to moderate the relationship between maternal prenatal anxiety and offspring social‐emotional behaviors, including temperamental reactivity and externalizing problems from infancy through toddler age (Frigerio and Nazzari 2021; Grant et al. 2010; Thomas et al. 2017). While prior research suggests that maternal sensitivity and responsiveness can lead to better social development in offspring, how prenatal social engagement, which can support a caregiving environment, may interact to affect developing social‐emotional processes in offspring is unclear.

### 1.1 Social Brain Network

Social brain systems emerge in infancy, and can be important to both acquisition of social competencies and trajectories of social‐emotional development (Grossmann and Johnson 2007; Ilyka et al. 2021; Mcpartland and Pelphrey 2012). Additionally, a plethora of research has demonstrated the importance of maternal caregiving behavior, such as sensitivity, for neurocognitive development, particularly related to social‐emotional competence (Curley and Champagne 2016). For example, children with larger neonate total brain volume demonstrated better cognitive outcomes when exposed to higher maternal sensitivity in infancy, and poorer cognitive outcomes when exposed to lower maternal sensitivity, reflecting differential susceptibility to maternal sensitivity at 6 months in relation to future cognitive development (Nolvi et al. 2020). At 6 months of age, maternal sensitivity has been positively associated with functional connectivity between the hippocampus and other brain areas critical to emotional regulation and socio‐emotional functioning (Rifkin‐Graboi et al. 2015), and lower maternal sensitivity in early infancy predicted development of neuroanatomy related to learning and stress regulation in childhood, such as larger amygdalae in males six years of age (Lee et al. 2019). Fewer studies have considered prenatal maternal social environments and offspring neurodevelopment. One study demonstrated that partner status of mothers moderated the relationship between socioeconomic status and neonate brain volumes, in that infants with mothers with lower SES and a partner had greater brain volumes in areas such as the middle temporal and occipital, inferior frontal and medial superior frontal regions in both hemispheres, and the anterior cingulate of the left hemisphere compared to infants whose mothers did not have a partner (Spann et al. 2020). Similar patterns were seen in pregnant women with higher SES who did not have a partner compared to those with a partner. Further research is needed to understand how both prenatal and postnatal social and caregiving environments and behaviors interact with offspring neurodevelopment across infancy.

### 1.2 The Current Study

There remains a gap in understanding how prenatal social support may shape postnatal maternal caregiving behavior and bonding during dyadic interactions with their infants, and in turn how this supports social‐emotional development of the offspring. The aim of this study was to assess associations between social‐emotional behaviors in both mothers and infants longitudinally, across the prenatal period through 14 months postnatal. The associations between prenatal social support, maternal sensitivity at 4 months, infant social skill and language development at 4 months, and attachment security at 14 months of age are considered through multiple pathways to assess the complex interrelationships that may exist between these social variables. We hypothesized that mothers who endorsed higher satisfaction of social support during pregnancy, as well as those who demonstrated higher sensitivity in caregiving behavior postnatally, would have infants who demonstrated increased social development at 4 months of age, which would subsequently lead to a more secure attachment at 14 months.

## 2 Methods

### 2.1 Participants

Pregnant individuals ages 14 to 21 years old were recruited through the Department of Obstetrics and Gynecology at Columbia University Irving Medical Center (CUIMC), Weill Cornell Medical College, and flyers posted throughout the CUIMC area as part of a prospective study examining pregnancy behaviors in adolescents and young adults and their infant outcomes through 14 months of age from 2009 to 2012. Pregnant individuals had no major health problems at the time of recruitment and received routine prenatal care. Pregnant individuals were excluded if they used recreational drugs, tobacco, alcohol, or medications that affect cardiovascular functions, or lacked fluency in English. Parental consent and adolescent assent were obtained for pregnant adolescents to participate in the study. Pregnant individuals aged 18 and older provided written informed consent for themselves, and mothers provided written informed consent for their infants to participate in the study. All study procedures were approved by the New York State Psychiatric Institute Institutional Review Board.

### 2.2 Measures

#### 2.2.1 Prenatal Social Support

The Social Support Questionnaire (SSQ) was completed during the second or third trimester of pregnancy. The SSQ is a 27‐item self‐report questionnaire that assesses the quality of social support and perceived satisfaction with that support. Each of the 27 questions is broken into a two‐part answer: The first part asks participants to list all the people that fit the description of the question, while the second part asks participants to indicate how satisfied they are, in general, with the people they listed in part 1. For part 1, the number of individuals reported across each question is totaled. An individual person is only counted once even if they are reported for more than one question. In part 2, participants rate their satisfaction on a scale from 1 (very dissatisfied) to 6 (very satisfied). To compute the total satisfaction score, scores are summed and then divided by the total number of items, resulting in a total average score ranging from 1 to 6. Total average satisfaction of social support scores and total number of social support persons were used in analyses. There were 91 maternal participants who had corresponding follow‐up data and were used in analyses.

#### 2.2.2 Neonate Social Brain Network

##### 2.2.2.1 Imaging Procedures

A subset of infants was imaged within the first 6 weeks of postnatal life (postmenstrual age [PMA] ≤ 46 weeks). Infants were fed, swaddled, and acclimated to the scanning environment and scanner noise by listening to a tape recording of the scanner sounds played prior to each pulse sequence. Before the start of each sequence, infants were given time to fall asleep without using sedatives while lying on the scanner bed. Infants wore foam and wax ear plugs and ear shields to dampen scanner noise (Natus Medical Inc., San Carlos, CA). MRI‐compatible electrocardiogram (EKG) leads were placed on the infant's chest, and a pulse oximetry sensor was placed on the infant's toe. Heart rate and oxygen saturation were continuously monitored during the scan (InVivo Research, Orlando, FL).

##### 2.2.2.2 Imaging Parameters

Images were obtained using a 3 Tesla General Electric (GE) Signa MRI scanner (Milwaukee, Wisconsin) and an 8‐channel head coil. High resolution anatomical T2‐weighted images were acquired using a 2D, multiple‐shot, fast spin echo pulse sequence that employed periodically rotated overlapping parallel lines with enhanced reconstruction (PROPELLER) to reduce motion artifacts in reconstructed MR images [18]: repetition time (TR) = 10,000 ms; echo time (TE) = 130 ms; echo train length (ETL) = 32; matrix size = 192 × 192; field of view (FOV) = 190 × 190 mm; phase FOV = 100%; slice thickness = 1.0 mm; number of excitations (NEX) = 2. The spatial resolution of the T2‐weighted images was 1 mm^3^. Functional images were acquired using a standard echo‐planar imaging sequence: TR = 2200 ms; TE = 30 ms; matrix size = 64 × 64; FOV = 190 × 190 mm; phase FOV = 100%; slice thickness = 5.0 mm, contiguous; number of slices = 24; bandwidth = 7812.5 Hz. Although the number of runs acquired varied per participant due to compliance, a median of 22 min 26.4 s of usable data across all runs was obtained for each infant.

##### 2.2.2.3 Common Space Registration

First, anatomical images were skull stripped using FSL (https://fsl.fmrib.ox.ac.uk/fsl/) and any remaining non‐brain tissue was manually removed. All further analyses were performed using BioImage Suite (Joshi et al. 2011) unless otherwise specified. Anatomical images were linearly aligned to a single infant anatomical scan from an independent study (Scheinost et al. 2016) using a 12 parameter affine registration by maximizing the normalized mutual information between images. Next, anatomical images were non‐linearly registered to an evolving group average template in an iterative fashion using a previously validated algorithm (Scheinost et al. 2017). This algorithm iterates between estimating a local transformation to align individual brains to a group average template and creating a new group average template based on the previous transformations. The local transformation was modeled using a free‐form deformation (FFD) parameterized by cubic B‐splines. This transformation deforms an object by manipulating an underlying mesh of control points. The deformation for voxels in between control points was interpolated using B‐splines to form a continuous deformation field. Positions of control points were optimized using a conjugate gradient descent to maximize the normalized mutual information between the template and individual brains. After each iteration, the quality of the local transformation was improved by increasing the number of control points and decreasing the spacing between control points to capture a more precise alignment. A total of 5 iterations were performed with decreasing control point spacings of 15 mm, 10 mm, 5 mm, 2.5 mm, and 1.25 mm. To help prevent local minimums during optimization, a multi‐resolution approach was used with three resolution levels at each iteration. Finally, functional images were rigidly aligned to the anatomical images.

All transformation pairs were calculated independently and combined into a single transform, warping the single participant results into common space. This single transformation allows the individual participant images to be transformed to the common space with only one transformation, thereby reducing interpolation error.

##### 2.2.2.4 Connectivity Processing

Motion correction was performed using SPM8 (http://www.fil.ion.ucl.ac.uk/spm/). Images were warped into 3 mm^3^ common space using the non‐linear transformation and cubic interpolation. Next, images were iteratively smoothed until the smoothness of an image had a full‐width half maximum of approximately 8 mm using AFNI's 3dBlurToFWHM (http://afni.nimh.nih.gov/afni/). Iteratively smoothing to a set smoothness reduces the smoothing applied to the image (which helps resolve smaller structures like the amygdala) and motion‐related confounds. Several covariates of no interest were regressed from the data, including linear and quadratic drifts, mean cerebral‐spinal‐fluid (CSF) signal, mean white‐matter signal, and mean gray matter signal. For additional control of possible motion‐related confounds, a 24‐parameter motion model (including six rigid‐body motion parameters, six temporal derivatives, and these terms squared) was regressed from the data. The functional data were temporally smoothed with a Gaussian filter (approximate cutoff frequency = 0.12 Hz).

##### 2.2.2.5 Motion Analysis

As motion and amount of data for analysis affect functional connectivity measures (Noble et al. 2017; Van Dijk et al. 2012), we employed a strict inclusion criterion that participants had at least 2 runs of data with an average frame‐to‐frame motion of less than 0.1 mm. For infants with more than 2 runs with good movement, we selected the 2 runs with the least frame‐to‐frame motion. We detected no significant correlations between motion and maternal experience of discrimination and acculturation (r's < 0.15, *p*’s > 0.4). Further, as described above, we employed global signal regression, a 24‐parameter motion model regression, and uniform smoothing to minimize motion confounds not accounted for by our inclusion criteria.

##### 2.2.2.6 Seed Connectivity

After preprocessing, we assessed whole‐brain seed to voxel connectivity across five bihemispheric brain regions associated with the social brain network. Brain regions included the medial prefrontal cortex, inferior frontal gyrus, superior temporal lobe, temporal pole, and the posterior cingulate cortex (Figure S1). These areas were chosen based on their role in social behavior. The medial prefrontal cortex circuits have been implicated in social cognition, such as social decision‐making and affective processes (Gangopadhyay et al. 2021; Gordon et al. 2011; Huang et al. 2020). The inferior frontal gyrus plays a role in social touch (Peled‐Avron et al. 2019). The superior temporal lobe and temporal pole, particularly the superior temporal sulcus, support social cognition and perception, as well as face perception, a socially important process (Barton 2022; Pelphrey and Carter 2008; Smith et al. 2023). The posterior cingulate cortex may play a role in social‐emotional information processing (Leung and Lau 2020), and is important for regulation of attention between internal and external thoughts and self‐referential thought, both of which can impact social behavior (Brewer et al. 2013; Leech et al. 2011; Leung and Lau 2020). Additionally, these areas have all been implicated in a network for theory of mind, or mentalizing, a crucial skill for social cognition (Poulin‐Dubois 2020; Yang et al. 2015). The time course of the reference regions in each participant was then computed as the average time course across all voxels in the seed regions. These time courses were correlated with the time course for every seed to create r‐values, reflecting seed‐to‐seed connectivity. These r‐values were transformed to z‐values using Fisher's transform and averaged, yielding a single value representing the strength of connectivity in the social brain for each participant. A total of 43 neonates had fMRI data used in analyses.

#### 2.2.3 Maternal Sensitivity

Maternal sensitivity was observationally coded from videotapes of mother‐child free play when the child was 4 months of age using well‐validated coding schemes for maternal warmth and responsiveness. Global ratings of warmth and responsiveness used a 5‐point scale: 1 = almost never, 2 = some of the time, 3 = half the time, 4 = most of the time, and 5 = almost always and were rated at the end of the video. Ratings of warmth were based on evidence from indicators like positive affect, praise, encouragement, physical affection, and acceptance of the child. Ratings of responsiveness were based on actions of the mother such as consistent involvement, timely and appropriate responses to the child's signals, and following the child's lead. The rating scales were adapted from well‐validated scales widely used in previous research, and were chosen due to their relevance to the construct of sensitivity (e.g., Landry et al. 2006, 2001; Merz et al. 2015). Ratings demonstrated good internal consistency across ages with Cronbach's alphas between .81 and .91 and prior interrater reliability within good ranges (r = 0.74) (Hallgren 2012; Landry et al. 2006, 2001). Consensus codes of warmth and responsiveness were totaled and used in analyses. A total of 72 maternal participants had warmth + responsiveness total scores that were used in analyses.

#### 2.2.4 Infant Social Development

The Bayley Scales of Infant and Toddler Development, Third Edition (Bayley, 2006; BSID‐III) was administered at 4 months of age. The BSID‐III is a standardized assessment of developmental functioning in early childhood between 1 and 42 months. It assesses five domains of development: cognition, language, motor, adaptive behavior, and social‐emotional.

##### 2.2.4.1 Language Development

The language component of the BSID‐III has both a receptive and expressive language scale. Scores from the expressive and receptive language scores are totaled to create a language sum score. This sum score is transformed into a standardized language composite score. This composite score for language was then used in analyses. A total of 83 infant participants had language composite scores at 4 months of age that were used in analyses.

##### 2.2.4.2 Social Skills Development

The adaptive behavior caregiver‐report questionnaire assesses the child's functional skills and ability to adapt to demands of daily living activities in multiple domains, including social skills. The social skills scaled score from this adaptive behavior questionnaire was used. Social skills scaled scores range from 1 to 19, with 8‐12 considered average. A total of 72 infant participants had social skills scaled scores from this questionnaire at 4 months of age used in analyses.

#### 2.2.5 Infant Attachment Security

The Strange Situation is a standardized procedure developed to observe attachment security in children 9 to 24 months within the context of caregiver relationships. It takes approximately 30 min to administer and was videoed when the children were 14 months of age. Trained coders reviewed the videos of the Strange Situation and classified the child into one of four patterns of attachment security: (A) secure, (B) insecure avoidant, or (C) insecure resistant. Children are rated/considered on interactive behaviors of proximity‐ and contact‐seeking, contact‐maintaining, resistance, and avoidance. These interactive behaviors are used to determine attachment security classification. A consensus for classification of attachment security pattern was reached between research assistants, and those consensus classifications were used for analyses. For our analyses, insecure avoidant and insecure resistant classifications were combined into one insecure group. A total of 56 infant participants had usable attachment classification coding for analyses.

### 2.3 Statistical Analyses

Descriptive statistics were conducted in SPSS 29.0 (IBM). We also conducted correlations, t‐tests, and one‐way analysis of covariance between demographic variables and maternal prenatal social support, 4 month maternal sensitivity, and 4 month infant social skill and language development to determine any covariates.

Using MPlus 8.4 (Methuen and Methuen), we then used path analysis with maximum likelihood to account for missing data to assess the direct effects of prenatal social support on maternal sensitivity (warmth + responsiveness), infant social development (social skills and language) at 4 months, and infant attachment security classification at 14 months. Direct effects between maternal sensitivity and infant language and social development, as well as maternal sensitivity, infant language development, and infant social skill development on attachment security classification at 14 months, were also tested. Additionally, this model tested indirect effects for (1) the concurrent associations of maternal sensitivity at 4 months with prenatal social support and infant social skill development at 4 months, and (2) the concurrent associations of maternal sensitivity with prenatal social support and infant language development at 4 months. Maternal perceived satisfaction with social support and the number of support persons given during the prenatal period were tested in separate models.

An exploratory path analysis was conducted to examine the influence of prenatal social support on neonate social brain network connectivity and neonate social brain network connectivity effects on maternal sensitivity and infant social‐emotional development at 4 months. Direct effects of prenatal social support and neonate social brain network connectivity, maternal sensitivity, and infant language and social skill development were tested, as were direct effects between neonate social brain network connectivity and maternal sensitivity, and infant social skill and language development.

## 3 Results

### 3.1 Descriptive Statistics

Table 1 displays demographic information for both mothers and infants. The mean age of pregnant individuals was 17.840 (SD = 1.259) years. The sample was a majority Hispanic/Latino. A total of 102 infants (male = 58, female = 44) were included in this study. Table 2 reports descriptive statistics for maternal and infant social variables prenatally to 14 months.

**TABLE 1:** Maternal and infant demographic information.

Variable	Mean (SD) / N (%)
Maternal	
*Age, yrs* (n = 321)	17.840 (1.259)
*Ethnicity* (n = 321)	
Hispanic/latino	281 (87.50)
Not hispanic/latino	40 (12.50)
*Primary language*	
English	215 (68.50)
Spanish	97 (30.90)
Other	2 (0.60)
*Household income* (n = 277)	
$0–$15,000	122 (44.00)
$16,000–$25,000	96 (34.70)
$26,000–$50,000	46 (16.60)
≥ $51,000	13 (4.70)
*Education level* (n = 318)	
≤ 12th grade	184 (57.86)
General education/high school diploma	116 (36.48)
Associates degree	2 (0.63)
Other	16 (5.03)
***Infant***	
*Sex* (n = 102)	
Male	58 (56.86)
Female	44 (43.13)
*Postmenstrual age at Scan, wk* (n = 45)	42.46 (1.71)
*Gestational age, wk* (n = 101)	39.29 (1.38)
*Birth weight, g* (n = 98)	3177.38 (503.93)
*Type of birth (n = 89)*	
Vaginal	72 (80.90)
Cesarean section	17 (19.10)

**TABLE 2:** Maternal and infant social variable descriptive statistics.

Variable	Mean (SD)
***Maternal***	
*Prenatal*	
Social support satisfaction (n = 294)	5.547 (0.688)
Number of social support persons (n = 294)	26.230 (3.088)
*4‐month*	
Warmth (n = 75)	3.293 (1.112)
Responsiveness (n = 75)	3.347 (1.020)
Sensitivity (warmth + responsiveness) (n = 75)	6.640 (1.908)
***Infant***	
*Neonate*	
Social brain network connectivity (n = 45)	0.093 (0.0419)
*4‐month*	
Social skill development (n = 75)	11.960 (2.362)
Receptive communication (n = 88)	9.375 (2.772)
Expressive communication (n = 89)	10.022 (2.335)
Language composite score (n = 88)	98.523 (10.848)
*14‐month*	
Attachment category (n = 60)	**N (%)**
Secure	34 (56.7)
Insecure	26 (43.3)

Bivariate associations were conducted between maternal demographic variables (e.g., maternal age) and independent and dependent variables. These variables included prenatal social support (satisfaction and number of persons), 4 month maternal sensitivity, and 4 month infant social development (social skills and language). There were no significant associations or differences found, which are reported in Table 3.

**TABLE 3:** Covariate analysis between maternal demographic characteristics and independent variable of interest.

	Maternal age r (p‐value)	Maternal ethnicity t (p‐value)	Maternal income F (p‐value)	Maternal education F (p‐value)
*Prenatal*				
Social support satisfaction	0.071 (0.224)	0.917 (0.365)	0.561 (0.691)	0.383 (0.682)
Number of social support persons	−0.029 (0.620)	0.362 (0.719)	0.308 (0.873)	1.395 (0.251)
*Neonate*				
Social brain network connectivity	0.012 (0.939)	−1.094 (0.308)	0.745 (0.533)	2.326 (0.119)
*4‐month*				
Maternal sensitivity (warmth + responsiveness)	0.054 (0.645)	0.709 (0.490)	1.336 (0.268)	2.070 (0.138)
Infant social skill development	0.157 (0.179)	−0.817 (0.430)	0.688 (0.634)	0.430 (0.653)
Infant language development	0.094 (0.384)	0.429 (0.674)	0.478 (0.792)	1.109 (0.337)

### 3.2 Satisfaction of Prenatal Social Support Models

**FIGURE 1:**
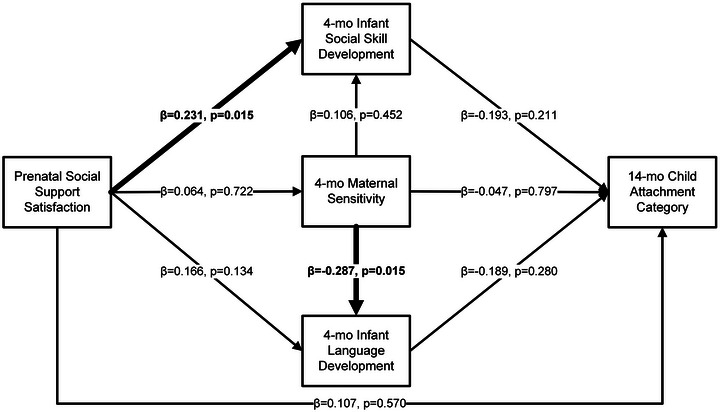
Associations between prenatal social support satisfaction, maternal sensitivity (warmth + responsiveness), child social skill, language, and attachment development.

The model (Figure 1) indicated a good fit (X^2^ = 0.001, *p* = 0.970; RMSEA < 0.001; CFI = 1.000; TLI = 1.000; SRMR = 0.001). Small to medium direct effects were found for maternal prenatal social support satisfaction predicting infant social skills at 4 months of age (β = 0.231, *p* = 0.015) and maternal sensitivity (warmth + responsiveness) on infant language scaled scores at 4 months of age (β = −0.287, *p* = 0.015). There were no significant indirect effects of maternal sensitivity at 4 months on the relationship between prenatal social support and infant social skills or infant language development in this model.

### 3.3 Number of Prenatal Social Support Persons Model

**FIGURE 2:**
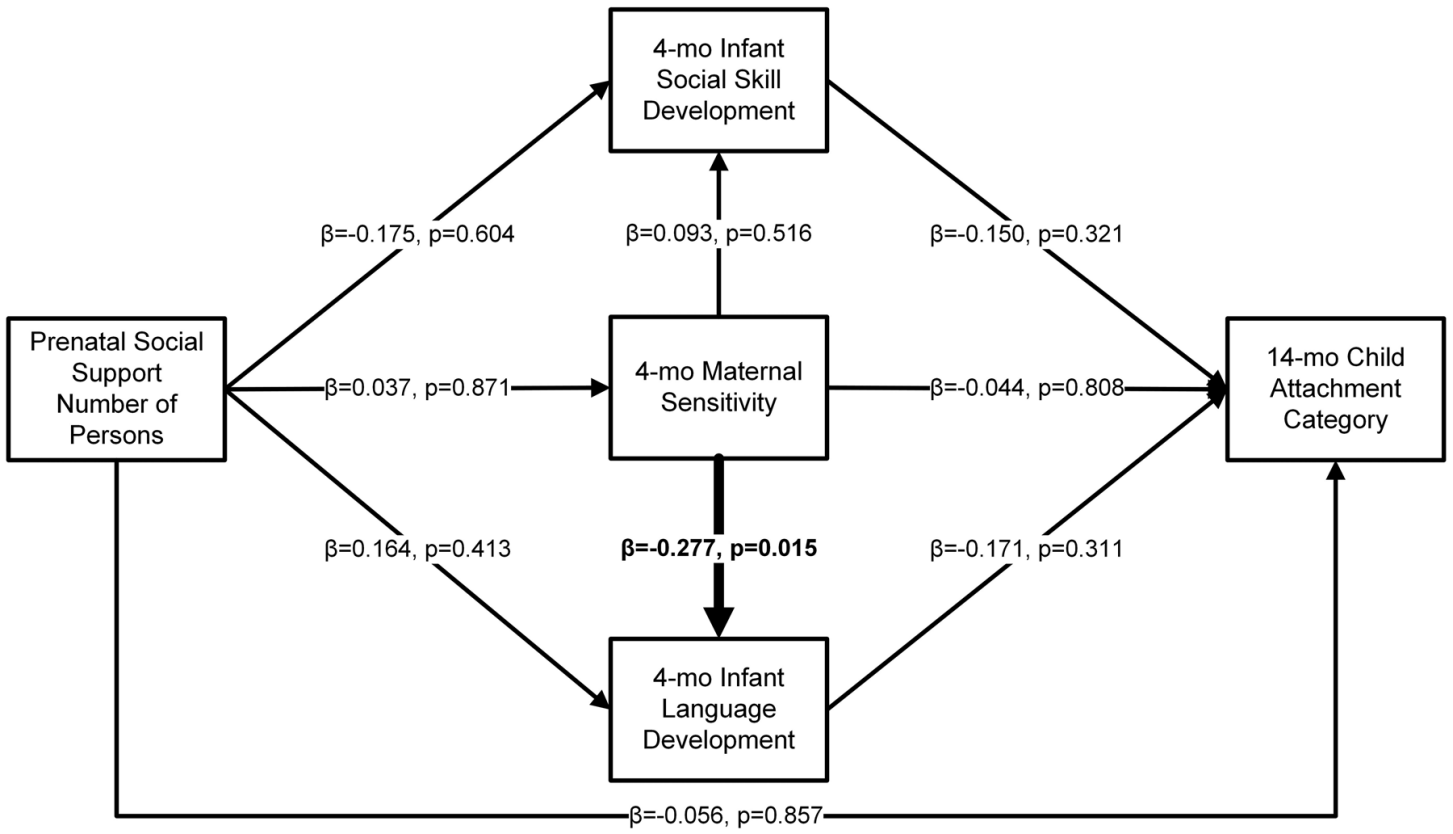
Associations between prenatal social support number of persons, maternal sensitivity (warmth + responsiveness), child social skill, language, and attachment development.

A model (Figure 2) testing the prenatal number of support persons with infant social skill and language development and maternal sensitivity (warmth + responsiveness) at 4 months was conducted. This model indicated only small to medium direct effects of 4 month maternal sensitivity on 4 month language development (β = −0.277, *p* = 0.019), and continued to indicate good fit (X^2^ = 0.249, *p* = 0.618; RMSEA < 0.001; CFI = 1.000; TLI = 1.000; SRMR = 0.014). No indirect effects of maternal sensitivity were observed.

### 3.4 Exploratory Analyses With Social Brain Connectivity

**FIGURE 3:**
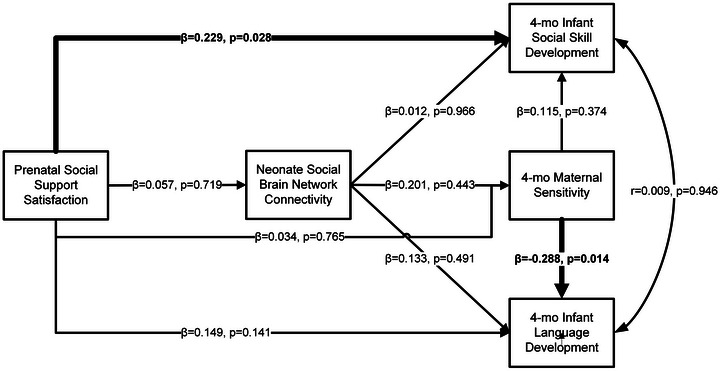
Associations between prenatal social support number of persons, neonate social brain network connectivity, maternal sensitivity (warmth + responsiveness), infant social skill, and language development.

As there were no significant associations of any variables with attachment classification at 14 month, this was removed from the social behavior model for exploratory testing (Figure 3). Direct and indirect effects for the mediation of social brain network connectivity on the relationship between prenatal social support satisfaction and infant social skill development at 4 months were tested. This model did not indicate a good fit (X^2^ = 0.000, *p* < 0.001), but continued to show direct effects of prenatal satisfaction of social support on 4 month infant social skill development and maternal sensitivity on infant language development at 4‐months. No other direct or indirect effects were significant.

## 4 Discussion

This study investigated associations between prenatal social support of the pregnant adolescent and maternal sensitivity and infant social‐emotional development, social skills, and language at 4 months postnatal, and child attachment security at 14 months of age. Additionally, associations of maternal and infant social‐emotional factors with neonate social brain network functional connectivity were explored. Overall, prenatal social support was associated with infant social skill development, and maternal sensitivity was associated with language development of the infant at 4 months of age.

### 4.1 Importance of Prenatal Social Support

Mothers who had higher satisfaction with their prenatal social support had infants that demonstrated higher levels of social skills at 4 months of age, while those mothers with more people in their support network did not. Additionally, the perceived quality of the social support received during pregnancy also appears more important to infant social development than how large or small a support network is. Perception of more satisfaction with current social support may provide the developing offspring with a more positive caregiving environment from the mother and others in utero and can be important to early prenatal bonding. For example, high or medium maternal social support can mitigate the relationship between an anxious romantic attachment and lower prenatal attachment (Ponti et al. 2021). Mothers who have good prenatal social support may continue to have this support after birth, and their newborns may be exposed to social situations and social dynamics more frequently. This exposure may provide opportunities for learning and practicing social skills within a larger group of people. These interactions would allow infants the opportunity to advance their social skills through increasingly fine‐tuned mutual adjustments within dyadic interactions that would result in improved prediction of the social world (Hoehl and Bertenthal 2021), and result in higher social skills in early infancy.

A lack of perceived support during pregnancy could have detrimental effects on mental health during pregnancy. Indeed, increased social support during pregnancy has been associated with a lower likelihood of depression and anxiety and can buffer its effects (Filippetti et al. 2022; Harrison et al. 2022; Modde Epstein et al. 2024; Tani and Castagna 2017). Mental health factors can impact how mothers and infants interact (Bernard et al. 2018; Graham et al. 2018), and can affect early attachment and infant social‐emotional development (Feldman et al. 2009; Filippetti et al. 2022; Norcross et al. 2017). Mothers who experience more satisfaction with their support while pregnant can experience positive buffering effects on their mental health prenatally that also continue postnatally (Modde Epstein et al. 2024; Tani and Castagna 2017), and, thus, mothers may be better able to promote their child's social development through refinement processes with maternal‐infant interactions (Hoehl and Bertenthal 2021). In addition to better understanding the continuity of the social support environment postnatally, maternal mental health, both prenatally and postnatally, will be important to consider in future studies to further understand possible interaction effects between social support and mental health that could influence maternal behavior and infant development.

Additionally, it is important to consider that mothers’ self‐reported both their perceived satisfaction with social support in pregnancy and their child's social skills at 4 months of age. Mothers’ reports may have reflected a positive bias in viewing other people in their life, which is a potential confound and may have affected the positive association found between maternal social support satisfaction and infant social skills. Additionally, this response bias may have affected the null association seen between prenatal social support and maternal sensitivity at 4 months postnatally, which was an observed measure. Future studies should consider using additional observed measures for maternal social support or infant social skill development to better understand this association.

### 4.2 Role of Maternal Sensitivity in Language Development

This study adds to literature supporting a link between maternal sensitivity and child language development. Prior research in the literature supports a positive effect of maternal sensitivity on child language development, even as early as 9 months (Borairi et al. 2021; Bornstein et al. 2020; Goldstein and Schwade 2008; Leigh et al. 2011; Madigan et al. 2019). In contrast, this study found that infants who were exposed to higher maternal warmth and responsiveness during play had lower language abilities. Several possibilities could account for this. First, a moderate level of responsiveness has been shown to be more effective for child development rather than higher amounts (Bornstein and Manian 2013). High levels of responsiveness may be intrusive or inappropriate, rather than optimally support the infants’ development. Over‐responsiveness can limit the development of self‐reliance and strain communication (Beebe et al. 2008; Bornstein and Manian 2013; van den Boom 1994). For example, if caregivers are immediately responsive towards their children's cues, children may not have opportunities to utilize or develop further skills to interact with the environment, such as communicating a want or indicating they understand a request. This could account for this relationship in this study. Second, both maternal sensitivity and infant language were measured at 4 months in this study. At this age, it may be developmentally appropriate for parents to provide a higher level of responsiveness to their young infants, and infant communication and language skills may be limited in their assessment. Third, infants in this largely Hispanic/Latino sample may have been exposed to more than one language within the first four months of life or resided in homes where the main language exposure was not English. Bilingual children can score lower on standardized language assessments that only assess one language, but are comparable to monolingual children when considering both language accomplishments (Hoff et al. 2012). Assessments for this study were conducted in English, and thus, lower scores from infants where this was the case may account for a negative association between maternal sensitivity and infant language development. Future studies should incorporate measures of child language at later ages and multiple languages to better understand the effects of maternal sensitivity in early infancy on child language development in diverse populations.

### 4.3 Emergence of Social Brain Network in Relation to Prenatal and Postnatal Factors

Prenatal social support does not appear to prime the neonate social brain network, nor does the neonate social brain network impact maternal or infant social behavior at 4 months. While social networks emerge in infancy (Mcpartland and Pelphrey 2012), the young age of the neonates in this study may have limited the relative strength of connections between social areas, as they were limited in exposure to complex postnatal social environments. Rapid neurodevelopment and continued exposure to social environments may continue to contribute to the development of the social brain network. A growing body of literature demonstrates the impact of postnatal maternal sensitivity on offspring neurodevelopment, particularly functional connectivity, in areas related to stress and social‐emotional regulation in infancy and into childhood (Copeland et al. 2022; Lee et al. 2019; Rifkin‐Graboi et al. 2015). Additionally, early neurodevelopment can play a role in susceptibility to social environmental exposures (Nolvi et al. 2020). Given this, and as disruptions in early specialization of social areas of the brain can have cascading effects for neurodevelopment (Pelphrey et al. 2011), future studies should continue to probe the connectivity of social brain network areas across the full spectrum of infancy as exposure to complex social environments increases. This will provide the opportunity to investigate how variations in early caregiving environments prenatally and postnatally may impact and interact with offspring social‐emotional brain development longitudinally.

### 4.4 Strengths, Limitations, and Future Directions

This study has several strengths. This study includes the measurement of both maternal and offspring social factors across multiple timepoints from the prenatal period through 24 months of age for the offspring. This allowed more complicated statistical analysis to be used. The use of path analysis can aid in illuminating how these social‐emotional factors work together and the complex relationships that may exist between them. The inclusion of two maternal sensitivity measures at the same timepoint was unique and provided the opportunity to explore effects of different dimensions of sensitivity on infant social‐emotional development.

This study has several limitations. The sample size of this study was small and varied across social‐emotional factors, which may have impacted analysis. This was especially apparent with the small sample size of neonates with social brain network functional connectivity. The age of imaging data used to derive functional connectivity has a coarse resolution compared to current field norms, which may have affected our results in this area. Additionally, the sample was a majority Hispanic/Latino and comprised of adolescents and young adults, so findings may not be generalizable to other populations. Adolescent mothers have been found to be less sensitive and more intrusive during interactions with their children than adult counterparts (Firk et al. 2018; Krpan et al. 2005; Lee 2009), and these differences may have impacted this study. Future studies should include adult populations in pregnancy and larger, more diverse samples, particularly those with more current imaging data, to better understand social‐emotional processes between maternal‐offspring dyads over time.

## 5 Conclusions

This study supports the importance of maternal satisfaction with social support during pregnancy for infant social development. It adds to the evidence that supports a relationship between maternal sensitivity and child language development, and that maternal responsiveness is a critical component of sensitive behavior. Both social support during pregnancy and maternal responsiveness have served as mechanisms for intervention development (Baranek et al. 2015; Landry et al. 2008; Weis and Ryan 2012), and findings from this study support future intervention research with these modifiable targets to facilitate long‐term offspring social‐emotional development. Future studies should also engage larger and more diverse samples to better understand the intergenerational and bidirectional effects of maternal‐offspring social behaviors.

## Author Contributions

**Cristin M. Holland**: conceptualization, formal analysis, funding acquisition, methodology, project administration, software, visualization, writing – original draft, writing – review and editing. **Huili Sun**: data curation, formal analysis, writing – original draft, writing – review and editing. **Raimundo X. Rodriguez**: data curation, formal analysis, visualization, writing – original draft, writing – review and editing. **Margaret Bennett**: data curation, formal analysis, writing – review and editing. **Bin Cheng**: formal analysis, methodology, validation, writing – review and editing. **Bradley S. Peterson**: conceptualization, data curation, funding acquisition, investigation, methodology, resources, writing – review and editing. **Dustin Scheinost**: conceptualization, data curation, formal analysis, funding acquisition, methodology, resources, software, validation, visualization, writing – original draft, writing – review and editing. **Marisa N. Spann**: conceptualization, data curation, formal analysis, funding acquisition, investigation, methodology, project administration, resources, software, supervision, validation, writing – review and editing.

## Conflicts of Interest

The authors declare that they have no known competing financial interests or personal relationships that could have appeared to influence the work reported in this paper.

## Funding

This work was supported by the Eunice Kennedy Shriver National Institute for Child Health and Human Development K23HD092589 (MS); National Institute of Mental Health [grant numbers R01MH093677 (BSP), K24MH127381 (MS), and R01MH126133 (MS & DS)]; and the National Center for Advancing Translational Sciences [grant number TL1TR001875 (CMH)].

## Ethics Statement

Study procedures were approved by the Institutional Review Board of the New York State Psychiatric Institute/Columbia University Medical Center, and all participants provided written informed consent.

## Supporting information




**Supplementary Figure**: brb371059‐sup‐0001‐FigureS1.jpeg

## Data Availability

Data sharing is not applicable to this article as no new data were created or analyzed in this study. Data are not available as the consent form at the time of the study did not have the provision for this.
