# Supplementary figures and images for: Associations of Prenatal Social Support in Adolescent Mothers With Measures of Social‐Emotional Development in Their Young Children
Source: Brain Behav. 2025 Nov 17;15(11):e71059. doi: 10.1002/brb3.71059 (PMC12623451; doi:10.1002/brb3.71059)

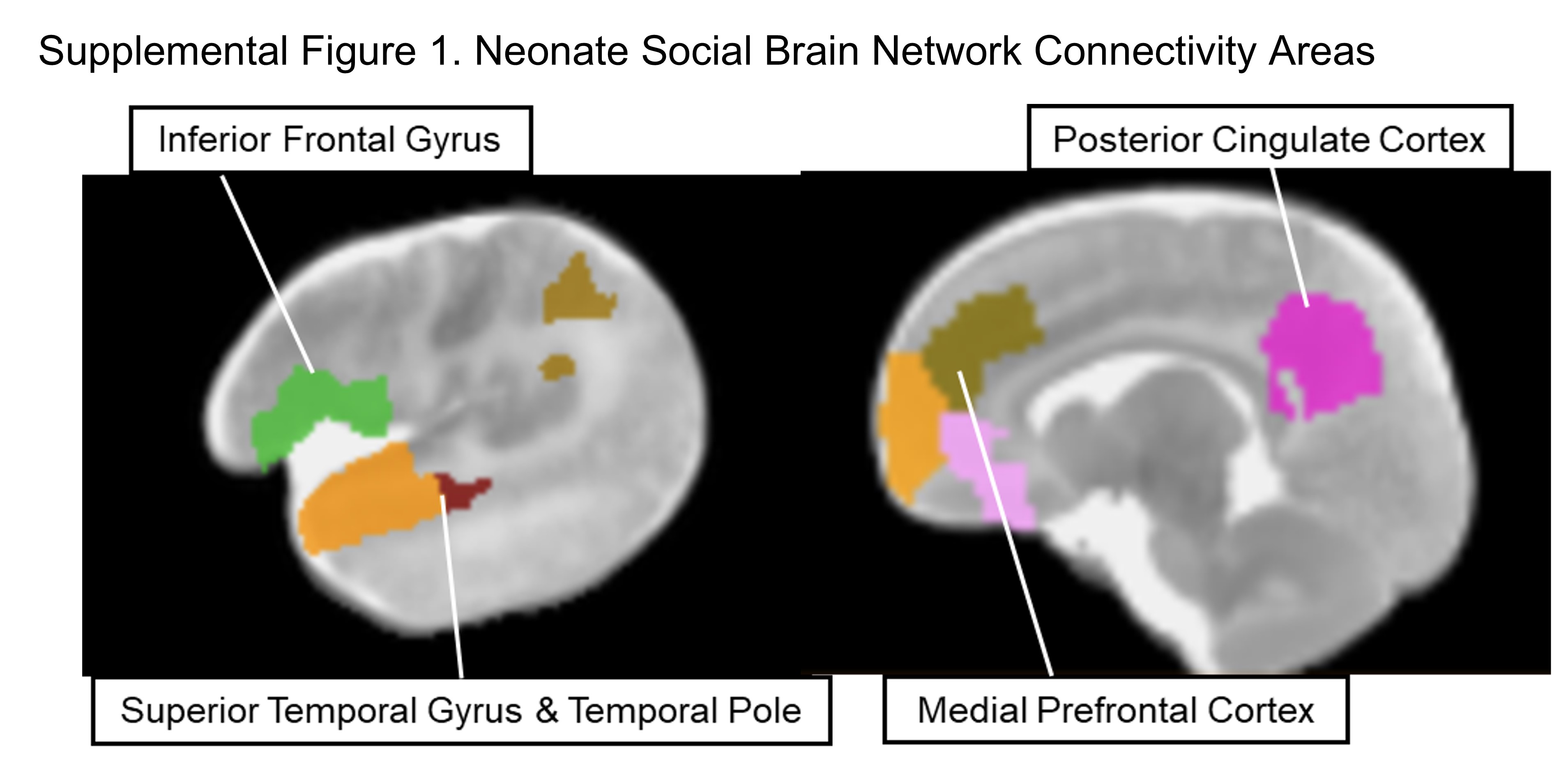

Supplement: Supplementary file 1 — Supplementary Figure: brb371059‐sup‐0001‐FigureS1.jpeg [file BRB3-15-e71059-s001.jpeg]
